# Development of a Novel Test for Simultaneous Bacterial Identification and Antibiotic Susceptibility

**DOI:** 10.1155/2016/5293034

**Published:** 2016-10-30

**Authors:** Jonathan Faro, Malika Mitchell, Yuh-Jue Chen, Sarah Kamal, Gerald Riddle, Sebastian Faro

**Affiliations:** ^1^The Woman's Hospital of Texas, 7400 Fannin Suite 930, Houston, TX 77054, USA; ^2^The University of Texas Health Science Center at Houston, Medical College, Houston, TX 77054, USA

## Abstract

*Background*. Elucidation of a pathogen's antimicrobial susceptibility requires subculture after the organism is first isolated. This takes several days, requiring patients to be treated with broad-spectrum antibiotics. This approach contributes to the development of bacterial resistance.* Methods*. Microtiter wells were coated with a polyclonal antibody targeting the pathogen of interest. Bacterial suspensions were added in the presence/absence of selected antibiotics. After washing, captured bacteria were detected.* Findings*. Group B streptococcus (GBS),* Enterococcus faecalis*, and* Neisseria gonorrhoeae* were each detected at 10^5^ bacteria/mL following a 20-minute incubation period. Susceptibility to select antibiotics was discernable following a 6-hour incubation period (GBS and* Enterococcus*). Sensitivity was increased to 10^−2^ bacteria/mL for GBS, 10^−1^ bacteria/mL for* E. faecalis*, and 10^1^ bacteria/mL for* N. gonorrhoeae* following 18–24-hour culture.* Conclusion*. This novel assay allows for the highly sensitive and specific identification of a pathogen and simultaneous determination of its antimicrobial susceptibility in a reduced time.

## 1. Introduction

When confronted with a patient battling an infectious disease, elucidation of the offending pathogen requires great effort. Time is critical: broad-spectrum antibiotics are initiated after cultures are collected, as identification of the offending microbe requires 24–48 hours. To increase the sensitivity of culture, an enrichment step may be added [1]. The workup is not completed once a pathogen is identified: antimicrobial susceptibility must next be ascertained following additional subculture in the presence of select antibiotics for another 24–48 hours [2].

Several techniques have been developed to aid in reducing the time required to identify a pathogen. These techniques include the use of chromogenic agar/broth and nucleic acid amplification technology (NAAT) [3, 4]. While both of these techniques have been employed in screening for antenatal GBS colonization, the Centers for Disease Control and Prevention still recommend that culture be performed [5]. This is an absolute requirement for patients allergic to penicillin, as NAAT is not capable of providing antimicrobial susceptibility profiles. Additionally, the resources and highly specialized training required to perform NAAT are not universally available [6].

Recently, the Infectious Diseases Society of America released a paper detailing the current approach towards identifying clinical pathogens. Described is a process characterized as having inadequate sensitivity and significant time delays, thereby contributing to the development of greater and greater antimicrobial resistance [7]. These concerns are all too-well illustrated in our approach towards treatment for GBS prophylaxis, in which increasing resistance to penicillin has been observed, and resistance to clindamycin is on the rise [8, 9]. Additionally, there is concern that this practice will contribute to* E. coli* resistance observed in premature neonates [10]. Already, we have seen the implications of antimicrobial resistance with* Enterococcus*, and hospital acquired infections with this pathogen are estimated to add $27,000.00 per infection [11, 12].* N. gonorrhoeae* resistance has been increasing steadily, and multidrug resistance has recently been confirmed [13]. These three disparate organisms demonstrate a range in the microbial response to our directed approach to both prophylaxis and treatment.

Through a modification of a recently reported test, we have developed a method for the simultaneous identification of a pathogen and determination of its antibiotic susceptibility [14]. We have modified this test so that GBS,* E. faecalis*, and* N. gonorrhoeae* may be detected at dilute concentrations after 6-hour incubation. Additionally, we show that inducible resistance of GBS against clindamycin may be determined. Finally, we show that following an overnight incubation, test organisms may be detected at concentrations rivaling those published for PCR. As this test allows one to simultaneously identify a pathogen and determine its antimicrobial susceptibility, this novel technique provides a change in the clinician's approach to managing infectious diseases.

## 2. Methods

### 2.1. Bacterial Strains and Antibodies

Group B streptococcus clinical isolates 12386 and 01.12.76 were shown by disk diffusion to be susceptible or resistant to clindamycin, respectively.* Enterococcus faecalis* ATCC 29212 was confirmed to be susceptible to vancomycin by disk diffusion, and ATCC strain 51299 was confirmed to be resistant.* Neisseria gonorrhoeae* ATCC strain 31426 was shown to be resistant to penicillin. Strain 1279, a clinical isolate, was shown to be susceptible to penicillin.* E. coli, S. aureus, Candida albicans,* and Beta streptococcus groups A, C, F, and G were all clinical isolates.

Rabbit polyclonal anti-GBS antibody (1521), HRP-conjugated rabbit polyclonal anti-GBS antibody (1524), rabbit polyclonal anti-*Enterococcus* antibody (3711), and HRP-conjugated rabbit polyclonal anti-*Enterococcus* antibody (3714) were all obtained from Virostat (Portland, Maine). Rabbit polyclonal anti-*N. gonorrhoeae* antibody (PA1-7233) and HRP-conjugated rabbit polyclonal anti-*N. gonorrhoeae* antibody (PA1-73144) were purchased from ThermoFisher (Waltham, MA).

### 2.2. Bacterial Detection and Competition Experiments

96-well Immulon microtiter plates (ThermoFisher, Waltham, MA) were coated with specified antibody. For the GBS assay, anti-GBS antibody was first diluted 1 : 30 in bicarbonate buffer (Sigma, St. Louis MO). For the* N. gonorrhoeae* or* Enterococcus* assays, antibodies were diluted 1 : 200 or 1 : 100, respectively. 100 *μ*L of the antibody dilutions was placed in respective wells, and the plates were incubated at 4°C overnight. Wells were then washed three times with phosphate-buffered saline (PBS, Sigma Aldrich, St. Louis, MO) supplemented with 0.05% Tween-20 (Fisher Scientific, Pittsburg, PA). The plates were blocked for 30 minutes at room temperature with 200 *μ*L of StartingBlock*™* (ThermoScientific, Rockford, IL) followed by washing three times with PBS-Tw at room temperature.

Bacterial isolates were individually prepared at a concentration of approximately 10^8^ bacteria/mL based on 0.5 McFarland and confirmed by OD_600 _nm. Aliquots were plated on appropriate agar (Columbia colistin nalidixic acid blood agar, for GBS and* Enterococcus*, and Chocolate agar, for* N. gonorrhoeae*, Fisher Scientific, Pittsburg, PA), and colony forming units (CFUs) were determined following a 24–48-hour culture. Inoculates were prepared further in tenfold dilutions down to 10^−3^ bacteria/mL. For the Time Zero Test, isolates were diluted in PBS and incubated at room temperature for 20 minutes. For growth experiments, Fastidious Broth (Remel, Lenexa, KS) was substituted for PBS, and inoculates were diluted out in test tubes and incubated prior to being transferred to wells. Competing organisms were prepared at a concentration of 10^8^ bacteria/mL. Varying concentrations of inoculates were added to tubes containing an amount of competing organisms held steady at 10^8^ bacteria/mL in Fastidious Broth. After a prespecified incubation time-point (6 hours for antimicrobial susceptibility testing with GBS and* Enterococcus*, 9 hours for inducible resistance of GBS to clindamycin, or overnight for determining the limit of detection of all three organisms or determination of antimicrobial susceptibility with* N. gonorrhoeae*) organisms were transferred to microtiter wells and plates were then incubated for 20 minutes at room temperature. For the Time Zero assay, this transfer step was omitted, as inoculates were prepared directly in the wells. For growth experiments, 10 *μ*L of the incubated bacterial suspensions was added to 90 *μ*L of PBS in each well. Following 20-minute incubation, wells were washed three times with PBS. Horseradish peroxidase (HRP) conjugated antibody was next added in 100 *μ*L aliquots and plates were incubated for 10 minutes at room temperature. HRP-conjugated antibodies for all tests were diluted 1 : 100 in PBS.

After washing three times with PBS-Tw, TMB peroxidase substrate (KPL, Gaithersburg, MD) was added to the wells in 100 *μ*L aliquots. Plates were incubated for 3 minutes at room temperature. Reactions were terminated using Stop Solution (Thermo Scientific, Rockford, IL). Plates were read in an ELX 808 BioTek (Winooski, Vermont) plate reader at 450 nm.

## 3. Results 

### 3.1. Time Zero Test

**Figure 1:**
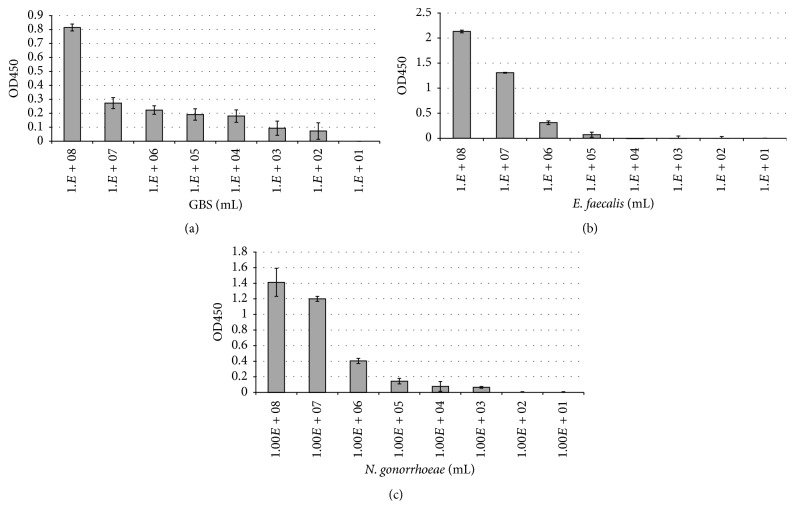
Determination of the limit of detection for the Time Zero Test. GBS (a),* Enterococcus* (b), and* N. gonorrhoeae* (c) were prepared at 0.5 McFarland in PBS and diluted out serially. Following 20-minute incubation at room temperature, wells were washed and then HRP-conjugated antibody directed against the bacterium of interest was used to detect any bound organism. Three distinct strains of GBS were tested, two strains of* Enterococcus,* and* N. gonorrhoeae* each. Results shown are of GBS clinical isolate 01.12.76 (a),* Enterococcus* ATCC strain 29212 (b), and* N. gonorrhoeae* strain 19424 (c); each study was performed in triplicate and representative of three individual experiments. Bacterial concentrations were confirmed by CFUs following plating on agar for 24–48 hours.

Serial dilutions of each bacterium were prepared starting with a 0.5 McFarland and diluted down to 10^1^ bacteria/mL in PBS. When processed on the pathogen-specific ELISAs, Group B streptococcus,* E. faecalis*, and* N. gonorrhoeae* each individually showed detection in the range of 10^2^–10^8^ bacteria/mL after 20-minute incubation (Figures 1(a)–1(c)). GBS was detected down to 10^2^ bacteria/mL,* E. faecalis* down to 10^5^ bacteria/mL, and* N. gonorrhoeae* down to 10^3^ bacteria/mL. In order to determine the specificity of the test, suspensions were tested in which the bacterium to be identified was diluted out, but the level of a competing bacterium was held constant at 10^8^ bacteria/mL in PBS. Testing of several commonly found vaginal cocolonizers mixed with the bacterium of interest revealed little to no interference with the assays (Supplemental Figures 1(A)–1(L) in Supplementary Material available online at http://dx.doi.org/10.1155/2016/5293034).

### 3.2. Limit of Detection

**Figure 2:**
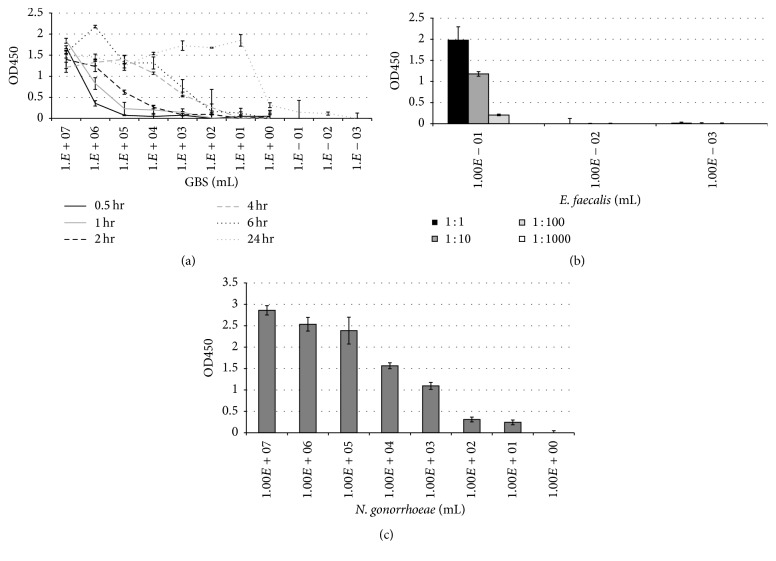
Determination of the limit of detection following incubation at 37° Celsius. GBS (a),* Enterococcus* (b), and* N. gonorrhoeae* (c) were prepared at 0.5 McFarland in Fastidious Broth and diluted out serially. For GBS, samples were incubated for either 0.5, 1, 2, 4, 6 or 24 hours at 37° Celsius (a). For* E. faecalis*, samples were incubated overnight at 37° Celsius. Tubes were noted to be turbid down to 10^0^ bacteria per mL, and this sample and the following two dilutions, 10^−1^ and 10^−2^ bacteria per mL, were prepared further by diluting in PBS out to 1 : 1,000. For* N. gonorrhoeae*, samples were prepared at a 0.5 McFarland in Fastidious Broth and then incubated overnight at 37° Celsius (c). Following these incubation time-points for each study, 10 *μ*L of each sample was transferred to microtiter wells containing 90 *μ*L PBS and allowed to stand for 20 minutes at room temperature. Any bound organism was detected by HRP-conjugated antibody against the specific pathogen. As with Figure 1, three distinct strains of GBS were tested, as were two strains of* Enterococcus* and* N. gonorrhoeae* each. Results shown are of GBS clinical isolate 01.12.76 (a),* Enterococcus* ATCC strain 29212 (b), and* N. gonorrhoeae* strain 19424 (c), each performed in triplicate and representative of three individual experiments. Again, bacterial concentrations were confirmed by CFUs following plating on agar for 24–48 hours.

Bacteria were next diluted in Fastidious Broth after first preparing a 0.5 McFarland. The ability to detect the test organism at greater dilutions increased when the incubation times were lengthened. GBS was detected down to 10^2^ bacteria/mL after 2-hour incubation, 10^1^ bacteria/mL after 6 hours, and 10^−2^ bacteria/mL after incubating overnight (Figure 2(a)).

A series of dilutions of* E. faecalis* starting at 0.5 McFarland was prepared similarly to that of GBS, and after incubating overnight (18 hrs) in Fastidious Broth, the tubes surrounding the turbidity point were assayed. Turbidity was observed down to 10^−1^ bacteria/mL. This tube was assayed directly, as well as was a series of dilutions of this sample. This same procedure was performed on the subsequent nonturbid dilutions, so that 10^−2^ and 10^−3^ bacteria/mL tubes were both assayed directly and then when diluted out further.* E. faecalis* was detected at 10^−1^ bacteria/mL, and when this sample was diluted out further, bacteria were capable of being detected following both 10-fold and 100-fold dilution (Figure 2(b)). In the nonturbid overnight culture tubes, no* E. faecalis* was detected (10^−2^ and 10^−3^ bacteria/mL).

*N. gonorrhoeae* was diluted out serially in Fastidious Broth, first starting with a 0.5 McFarland. After overnight incubation, the assay was run.* N. gonorrhoeae* was detected strongly at high concentrations, as well as at very low concentrations, 10^2^ and 10^1^ bacteria/mL (Figure 2(c)). The test was not capable of detecting bacteria less than 10^1^ bacteria/mL following 24-hour incubation.

### 3.3. Determination of Antimicrobial Susceptibility

**Figure 3:**
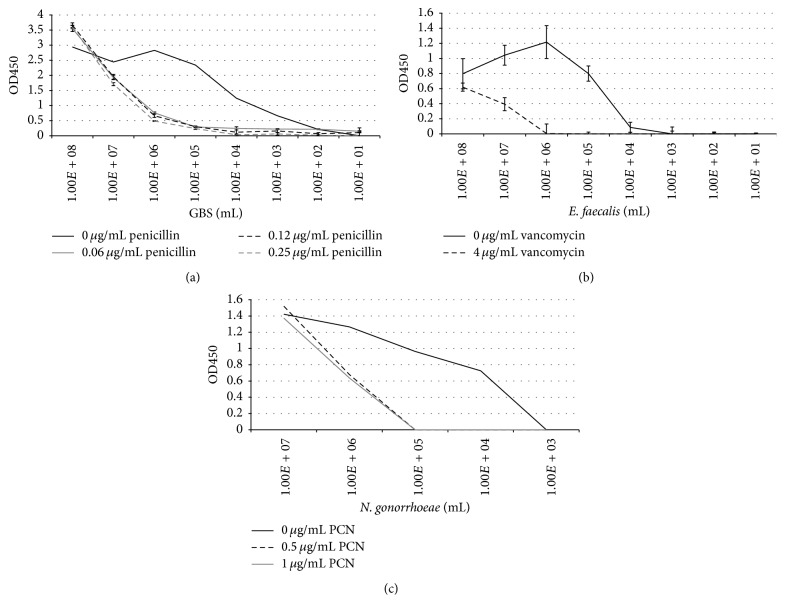
Bacterial identification and demonstration of susceptibility to selected antibiotics following a 6-hour culture. After first preparing a 0.5 McFarland in Fastidious Broth and then diluting bacterial isolates out, antibiotics were added to the wells giving the final concentrations indicated. (a) Dilutions of GBS strain 12386 (confirmed to be susceptible to penicillin by disk diffusion), in the presence of 0, 0.06, 0.12, or 0.25 *μ*g/mL penicillin. (b)* E. faecalis* was diluted out in Fastidious Broth in the presence (4 *μ*g/mL) or absence of vancomycin.* Enterococcus faecalis* ATCC strain 29212 was confirmed to be susceptible to vancomycin by disk diffusion. (c)* N. gonorrhoeae* was prepared and diluted out, as above, in 0, 0.5, or 1 *μ*g/mL penicillin. Strain 1279, a clinical isolate of* N. gonorrhoeae*, was shown to be susceptible to penicillin by disk diffusion. Each test was performed in triplicate and data shown are representative of three individual experiments. Again, bacterial concentrations were confirmed by CFUs following plating on agar for 24–48 hours.

Isolates were individually prepared in the presence or absence of selected antibiotics. After culturing for 6 hours, the assay was performed and susceptibility profiles were obtained. A strain of GBS sensitive to penicillin was cultured in the presence of increasing concentrations of the following antibiotics: penicillin, cefazolin, erythromycin, and clindamycin. At all concentrations of penicillin tested, growth of this strain of GBS was inhibited and no GBS was detected at 10^5^ bacteria/mL (Figure 3(a)). Incubation of GBS with increasing concentrations of cefazolin showed a dose effect, in which growth was observed at a more dilute concentration of antibiotic, 0.005 *μ*g/mL, but not when increasing amounts were used (Supplemental Figure  2(A)). Similar results were observed when the same strain was tested in the presence of either clindamycin or erythromycin (Supplemental Figures  2(B) and 3(C)).

Next, a GBS strain resistant to clindamycin but sensitive to erythromycin was prepared, as above. Supplemental Figure 2(D) shows that, at 0.25 *μ*g erythromycin, growth of the GBS strain was inhibited at 10^5^ bacteria/mL. Antibiotic concentrations were selected based on CLSI breakpoints. Conversely, at all concentrations of clindamycin tested, GBS was detected at levels similar to that of the no antibiotic control for this strain of GBS (Supplemental Figure  2(E), strain 165).

*Enterococcus faecalis* was tested by methods similar to that of GBS. When vancomycin sensitive* E. faecalis* was diluted out in the presence of vancomycin, no growth was detected at 10^5^ bacteria/mL (Figure 3(b)). When a vancomycin resistant strain was substituted under test conditions, however, this organism was detected at 10^5^ bacteria/mL after 6-hour incubation (Supplemental Figure  2(F)).

Strains of* N. gonorrhoeae* were prepared and diluted out as above, in the presence of either penicillin or ceftriaxone. Following 24-hour incubation, a strain of* N. gonorrhoeae* shown to be sensitive to penicillin was assayed. This strain was detected at 10^5^ bacteria/mL when tested in the absence of penicillin, but no bacteria were detected at this inoculum in wells which received penicillin (Figure 3(c)). When a penicillin-resistant strain of* N. gonorrhoeae* was tested, bacteria were strongly detected at 10^5^ bacteria/mL following 24-hour incubation in wells receiving less than 2 *μ*g/mL, with slight detection at 2 *μ*g/mL (Supplemental Figure  2(G)). A strain of* N. gonorrhoeae* sensitive to ceftriaxone was next tested and showed similar results: bacteria cultured in the absence of ceftriaxone were detected at 10^5^ bacteria/mL, but not when cultured in the presence of either 0.0625 *μ*g/mL or 0.125 *μ*g/mL ceftriaxone at this inoculum (Supplemental Figure  2(H)). Both antibiotic concentrations used were less than the recommended level of 0.25 *μ*g/mL in determining resistance to ceftriaxone [15].

### 3.4. Inducible Resistance of GBS against Clindamycin

GBS strains previously determined to show either noninducible (Isolate A) or inducible (Isolate B) resistance to clindamycin in the presence of erythromycin were tested following 9-hour incubation. In the noninducible resistance strain (Isolate A), GBS was detected only in wells which did not receive clindamycin (Table 1). When a strain capable of showing inducible resistance was tested, GBS was detected down to a 10^3^ bacteria/mL dilution, with very strong detection at 10^6^ bacteria/mL, moderate detection at 10^5^ bacteria/mL, and light detection at 10^4^ bacteria/mL.

## 4. Discussion

The development of microbial resistance to antibiotics has plagued contemporary clinicians. Following the introduction of each new antibiotic, resistant isolates have been detected, with resistance of* Staphylococcus* to Methicillin noted 2 yrs after the first use of this antibiotic in 1960, resistance of* Enterococcus* to vancomycin was noted 16 yrs after its introduction in 1972, and resistance of* Staphylococcus* to Linezolid was noted just 1 yr after its first use in 1996 [16]. The development of microbial resistance to an antibiotic has become the norm, and anticipated resistance has been confirmed again as recently as 2011 with the multidrug resistant* N. gonorrhoeae* strain reported in Japan [17].

That antimicrobial resistance has developed and continues to do so is not surprising. What is concerning is that the approach to managing patients with suspected infection/colonization has remained static, and in fact this approach continues to be reestablished, with the recent example of intrapartum GBS prophylaxis as recommended by the CDC in 2010 [5]. It is agreed that intrapartum GBS prophylaxis has led to a reduction in early-onset GBS sepsis: However, there is concern that this practice may promote resistant strains of* E. coli* [13]. The increased prevalence of vancomycin resistant* Enterococcus* in hospitalized patients is yet another example. As Enterococci are to a large degree resistant to cephalosporins, use of this class of antibiotic for surgical prophylaxis allows for overgrowth of* Enterococcus* at sites previously colonized by cephalosporin-sensitive organisms and may contribute to the increased number of hospital associated surgical site infections [18]. Perhaps nowhere is this battle between pathogen and microbicide more apparent than with* N. gonorrhoeae*. From the first use of sulfonamides in the 1940s, this bacterium has countered the development of resistant strains [19]. After showing that this pathogen is capable of developing resistance to any antibiotic directed against it, including sulfonamides, penicillin, tetracycline, spectinomycin, quinolones, macrolides, and now cephalosporins, it is apparent that novel approaches are needed beyond the two-drug counterattack recommended just last year [20].

We recently developed an immunoassay in which membranes coated with antibody against GBS are exposed to bacterial suspensions, and after incubating for a series of time-points, bound GBS is detected [14]. This assay was tested on patients' vaginal-rectal specimens and was found to have a high degree of sensitivity and specificity, with the added benefit of being capable of detecting nonbeta hemolytic streptococcal strains of GBS [21]. In order to decrease the interobserver variation seen with dot-blot assays, we converted the test to an ELISA format. In this study, we have substituted the anti-GBS antibodies for those directed against either* N. gonorrhoeae* or* Enterococcus*. We show that these polyclonal antibodies provide a great deal of sensitivity in a nonselective broth, with little interference from other commonly isolated cocolonizers Figure 1 and Supplemental Figure  1.

Following a six-hour incubation period, the limit of detection increased to 10^1^ bacteria per mL for GBS (Figure 2). Furthermore, we show that antibiotic susceptibility may be determined (Supplemental Figure  2). From this, one may envision a panel in which a series of wells are set up in which multiple clinically relevant antibiotics are tested, providing the clinician with a targeted approach to treatment in a greatly reduced timeframe (Figure 3).

A central facet of this assay involves cellular viability: the concentration of bacteria present in the well is related specifically to the incubation times utilized. By incubating overnight, the sensitivity of the test was increased significantly as live bacteria continued to grow and divide, with GBS being detected at 10^−2^ bacteria per mL,* Enterococcus* detected at 10^−1^ bacteria per mL, and* N. gonorrhoeae* detected at 10^1^ bacteria per mL (Figure 2). This allows for a sensitivity greater than published results for several PCR-based methods of detection following an enrichment step, with the unique advantage being that this test is capable of detecting viable cells [22–25].

This assay provides the unique approach of targeting the growth of a specific clinically relevant pathogen through the use of a capture antibody. An immediate binding assay demonstrates whether a pathogen is present or not. By then culturing the organism in the presence or absence of selected antibiotics, one may show that cells are viable and susceptible to specific antimicrobials, thereby allowing the clinician to then make an informed decision more rapidly.

## Supplementary Material

In Supplementary Figure 1, GBS (A-I), Enterococcus (J and K), or N. gonorrhoeae (L) were tested against sevaral commonly isolated cocolonizers. Test bacteria were diluted out, while competing organisms were held constant. In Supplemental Figure 2, GBS was shown to be sensitive to either cefazolin (A), clindamycin (B), or erythromycin (C) following a 6-hour incubation. Panels D and E show GBS resistance to clindamycin. Panel F shows Enterococcus, resistant to vancomycin. Finally, Panels G and H show N. gonorrhoeae resistant to penicillin or sensitive to ceftriaxone, respectively.

## Figures and Tables

**Table 1:** Detection of GBS following 9-hour incubation in the presence of erythromycin and dilutions of clindamycin. Isolate A (noninducible resistance to clindamycin) compared with isolate B (inducible resistance capable). ++++ OD > 3.0. +++ OD 2.0–2.9. ++ OD 1.0–1.9. + OD 0.1–0.9. − OD 0–0.099.

	10^7^ bacteria/mL		10^6^ bacteria/mL		10^5^ bacteria/mL		10^4^ bacteria/mL	
	Isolate A	Isolate B	Isolate A	Isolate B	Isolate A	Isolate B	Isolate A	Isolate B
Clindamycin 0 *μ*g/mL Erythromycin 1.0 *μ*g/mL	++++	++++	++++	++++	++++	++++	++++	+++

Clindamycin 0.5 *μ*g/mL Erythromycin 1.0 *μ*g/mL	−	++++	−	++	−	+	−	+

Clindamycin 0.05 *μ*g/mL Erythromycin 1.0 *μ*g/mL	−	++++	−	+++	−	+	−	+

Clindamycin 0.005 *μ*g/mL Erythromycin 1.0 *μ*g/mL	−	++++	−	+++	−	+	−	+
