# Supplementary material for: Development of a Novel Test for Simultaneous Bacterial Identification and Antibiotic Susceptibility
Source: Infect Dis Obstet Gynecol. 2016 Oct 30;2016:5293034. doi: 10.1155/2016/5293034 (PMC5107225; doi:10.1155/2016/5293034)
Supplement: Supplementary file 1 — In Supplementary Figure 1, GBS (A-I), Enterococcus (J and K), or N. gonorrhoeae (L) were tested against sevaral commonly isolated cocolonizers. Test bacteria were diluted out, while competing organisms were held constant. In Supplemental Figure 2, GBS was shown to be sensitive to either cefazolin (A), clindamycin (B), or erythromycin (C) following a 6-hour incubation. Panels D and E show GBS resistance to clindamycin. Panel F shows Enterococcus, resistant to vancomycin. Finally, Panels G and H show N. gonorrhoeae resistant to penicillin or sensitive to ceftriaxone, respectively. [file 5293034.f1.pdf]

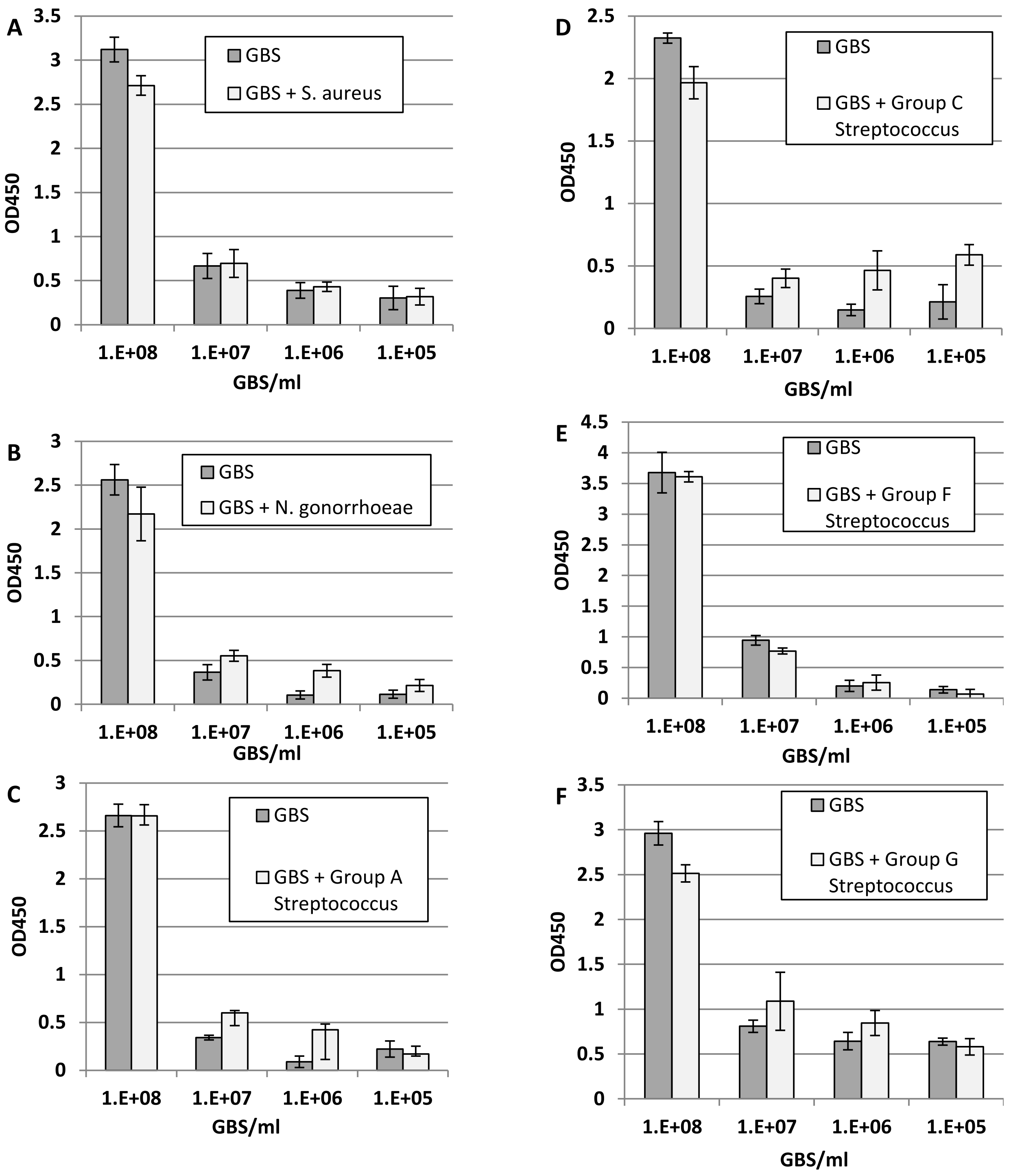
A
OD450
3.5
3
2.5
2
1.5
1
0.5
0
GBS
GBS + S. aureus
1.E+08
1.E+07
1.E+06
1.E+05
GBS/ml
B
OD450
3
2.5
2
1.5
1
0.5
0
GBS
GBS + N. gonorrhoeae
1.E+08
1.E+07
1.E+06
1.E+05
GBS/ml
C
OD450
3
2.5
2
1.5
1
0.5
0
GBS
GBS + Group A Streptococcus
1.E+08
1.E+07
1.E+06
1.E+05
GBS/ml
D
OD450
2.5
2
1.5
1
0.5
0
GBS
GBS + Group C Streptococcus
1.E+08
1.E+07
1.E+06
1.E+05
GBS/ml
E
OD450
4.5
4
3.5
3
2.5
2
1.5
1
0.5
0
GBS
GBS + Group F Streptococcus
1.E+08
1.E+07
1.E+06
1.E+05
GBS/ml
F
OD450
3.5
3
2.5
2
1.5
1
0.5
0
GBS
GBS + Group G Streptococcus
1.E+08
1.E+07
1.E+06
1.E+05
GBS/ml

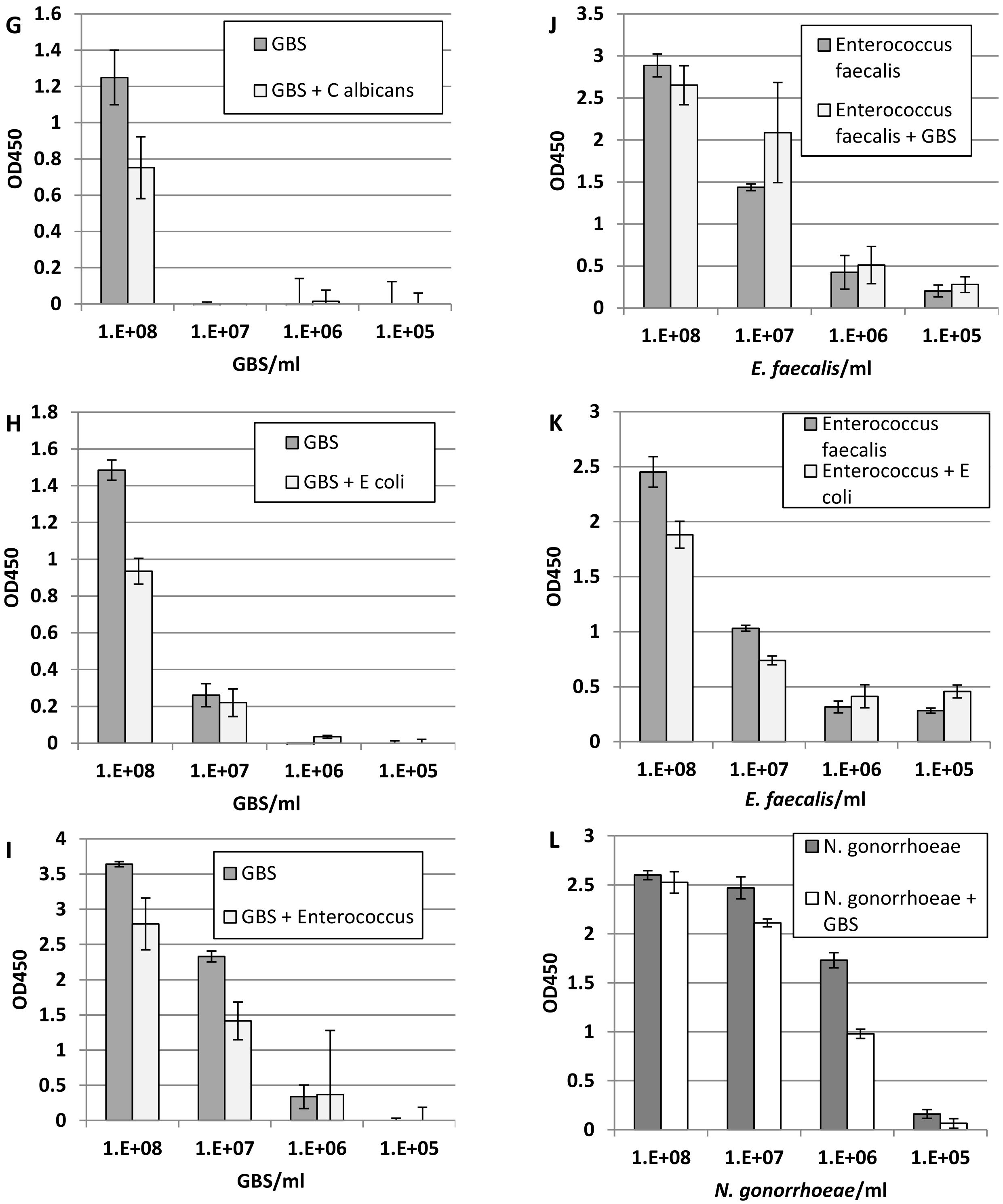
G
GBS
GBS + C albicans
OD450
0
0.2
0.4
0.6
0.8
1
1.2
1.4
1.6
1.E+08
1.E+07
1.E+06
1.E+05
GBS/ml
J
Enterococcus faecalis
Enterococcus faecalis + GBS
OD450
0
0.5
1
1.5
2
2.5
3
3.5
1.E+08
1.E+07
1.E+06
1.E+05
E. faecalis/ml
H
GBS
GBS + E coli
OD450
0
0.2
0.4
0.6
0.8
1
1.2
1.4
1.6
1.8
1.E+08
1.E+07
1.E+06
1.E+05
GBS/ml
K
Enterococcus faecalis
Enterococcus + E coli
OD450
0
0.5
1
1.5
2
2.5
3
1.E+08
1.E+07
1.E+06
1.E+05
E. faecalis/ml
I
GBS
GBS + Enterococcus
OD450
0
0.5
1
1.5
2
2.5
3
3.5
4
1.E+08
1.E+07
1.E+06
1.E+05
GBS/ml
L
N. gonorrhoeae
N. gonorrhoeae + GBS
OD450
0
0.5
1
1.5
2
2.5
3
1.E+08
1.E+07
1.E+06
1.E+05
N. gonorrhoeae/ml

Supplemental Figure 1 A-L. Detection of test bacteria in the presence of common co-colonizers. GBS (A-I), *E. faecalis* (J and K), and *N. gonorrhoeae* (L) were prepared at 0.5 McFarland in PBS and diluted out serially. In Panels A-I, dilutions of GBS were compared against samples in which the concentration of GBS was diluted out in the presence of a constant amount of a competing bacteria (held steady at 10^8 bacteria per ml). For panels J and K, dilutions of *E. faecalis* were compared against dilutions also containing either GBS (Panel J) or *E. coli* (Panel K), both held steady at 10^8 bacteria per ml. Panel L shows dilutions of *N. gonorrhoeae* compared with dilutions of the same organism, but with GBS held steady at 10^8 bacteria per ml in each sample. For Panels A-I, three distinct strains of GBS were tested, for panels J and K, two strains of *E. faecalis* were used and for Panel L, two strains of *N. gonorrhoeae* were utilized. Results shown are of GBS clinical isolate 01.12.76, (Panels A-I), *E. faecalis* ATCC strain 29212 (Panels J and K), and *N. gonorrhoeae* strain 19424 (Panel L). Each test was performed in triplicate and data shown are representative of three individual experiments. Bacterial concentrations were confirmed by CFUs following plating on agar for 24-48 hours.

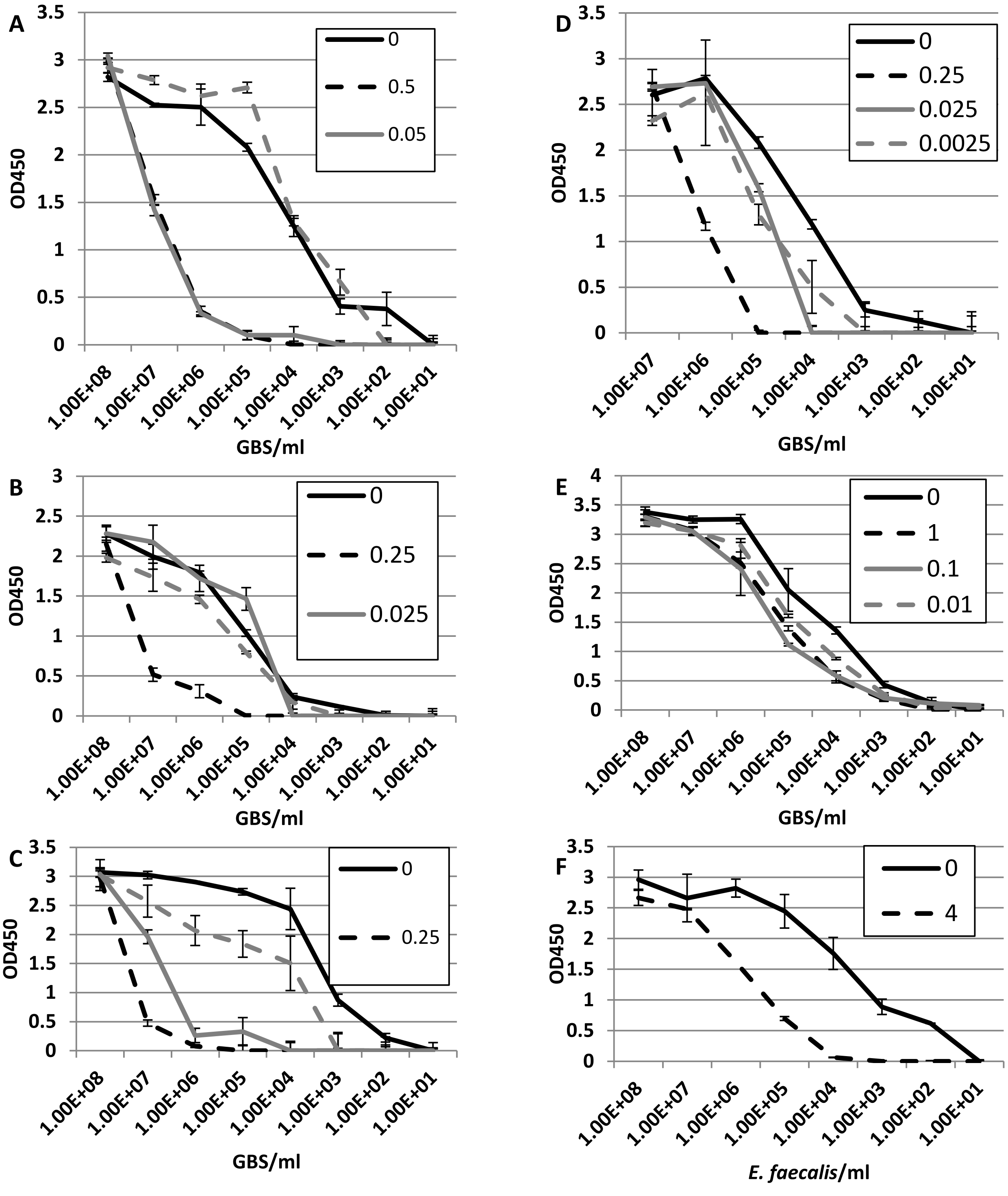

A
OD450
GBS/ml
0
0.5
0.05
B
0
0.25
0.025
C
0
0.25
D
0
0.25
0.025
0.0025
E
0
1
0.1
0.01
F
0
4
E. faecalis/ml

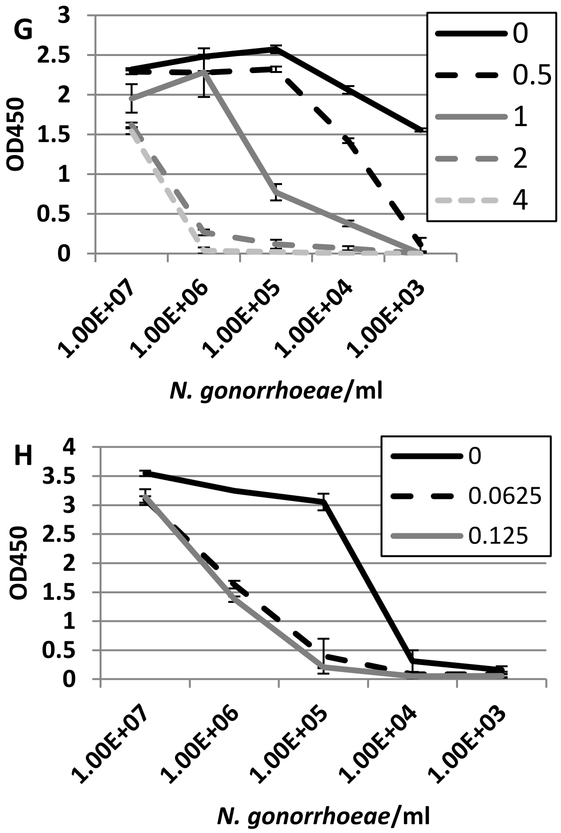
G
OD450
3
2.5
2
1.5
1
0.5
0
1.00E+07
1.00E+06
1.00E+05
1.00E+04
1.00E+03
N. gonorrhoeae/ml
0
0.5
1
2
4
H
OD450
4
3.5
3
2.5
2
1.5
1
0.5
0
1.00E+07
1.00E+06
1.00E+05
1.00E+04
1.00E+03
N. gonorrhoeae/ml
0
0.0625
0.125

Supplemental Figure 3 A-H. Demonstration of bacterial resistance to selected antibiotics.

After preparing a 0.5 McFarland in Fastidious Broth and then diluting bacterial isolates out, antibiotics were added to the wells giving the final concentrations indicated. A clinical isolate of GBS sensitive to penicillin (strain 12386) was diluted in the presence of either cefazolin at 0, 0.5, 0.05, or 0.005 μg/ml (Panel A), 0, 0.25, 0.025, or 0.0025 μg/ml clindamycin (Panel B), 0, 0.25, 0.025, or 0.0025 μg/ml erythromycin (Panel C). In Panels D and E, a clinical isolate of GBS sensitive to erythromycin but resistant to clindamycin was diluted in the presence of either 0, 0.25, 0.025, or 0.0025 μg/ml erythromycin (Panel D), or 0, 1, 0.1, or 0.01 μg/ml clindamycin (Panel E). Panel F shows a vancomycin-resistant strain of *E. faecalis*, strain 51299, diluted in either 0 or 4 μg/ml vancomycin. In Panel G, a penicillin-resistant strain of *N. gonorrhoeae*, strain 31426, was diluted in the presence of 0, 0.5, 1, 2, or 4 μg/ml penicillin. Finally, Panel H shows a ceftriaxone-sensitive strain of *N. gonorrhoeae*, strain 1279, diluted in the presence of 0, 0.0625 or 0.125 μg/ml ceftriaxone. Incubations were for 6 hours for all studies, except those involving *N. gonorrhoeae*, which were overnight. Each test was performed in triplicate and data shown are representative of three individual experiments. Bacterial concentrations were confirmed by CFUs following plating on agar for 24-48 hours.

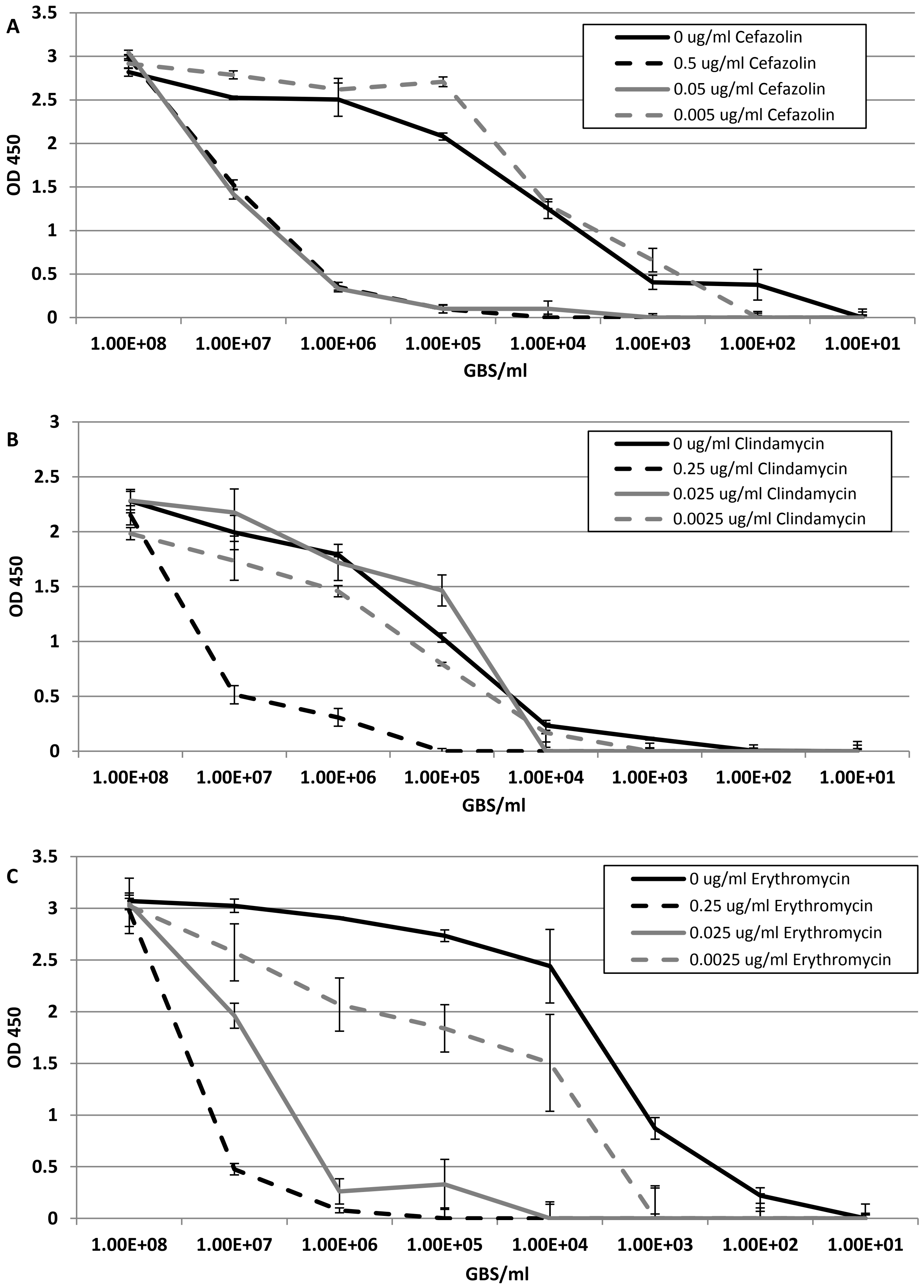
A
0 ug/ml Cefazolin
0.5 ug/ml Cefazolin
0.05 ug/ml Cefazolin
0.005 ug/ml Cefazolin
OD 450
GBS/ml
3.5
3
2.5
2
1.5
1
0.5
0
1.00E+08
1.00E+07
1.00E+06
1.00E+05
1.00E+04
1.00E+03
1.00E+02
1.00E+01
B
0 ug/ml Clindamycin
0.25 ug/ml Clindamycin
0.025 ug/ml Clindamycin
0.0025 ug/ml Clindamycin
OD 450
GBS/ml
C
0 ug/ml Erythromycin
0.25 ug/ml Erythromycin
0.025 ug/ml Erythromycin
0.0025 ug/ml Erythromycin
OD 450
GBS/ml

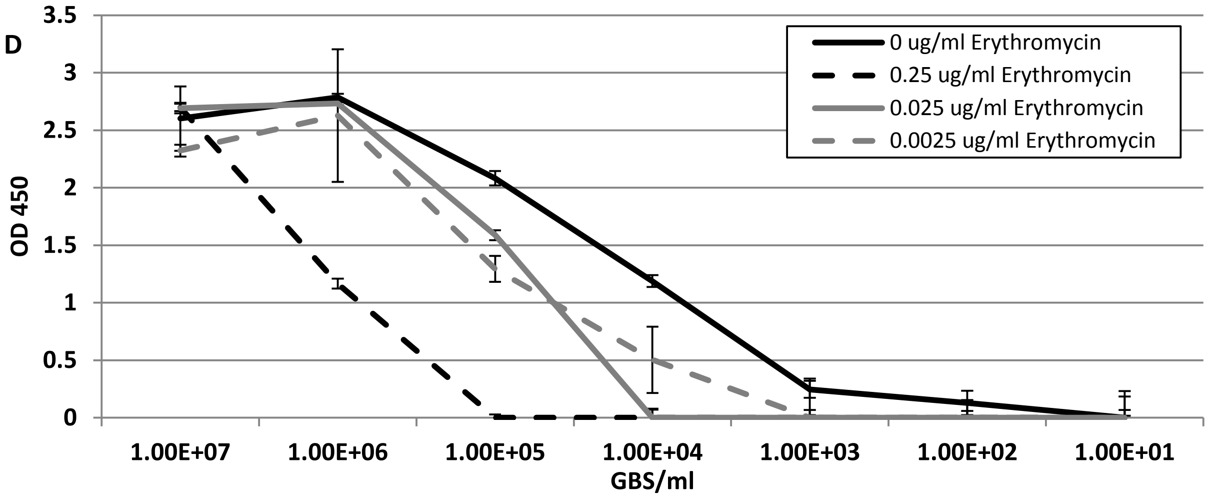
D
OD 450
3.5
3
2.5
2
1.5
1
0.5
0
1.00E+07
1.00E+06
1.00E+05
1.00E+04
1.00E+03
1.00E+02
1.00E+01
GBS/ml
0 ug/ml Erythromycin
0.25 ug/ml Erythromycin
0.025 ug/ml Erythromycin
0.0025 ug/ml Erythromycin

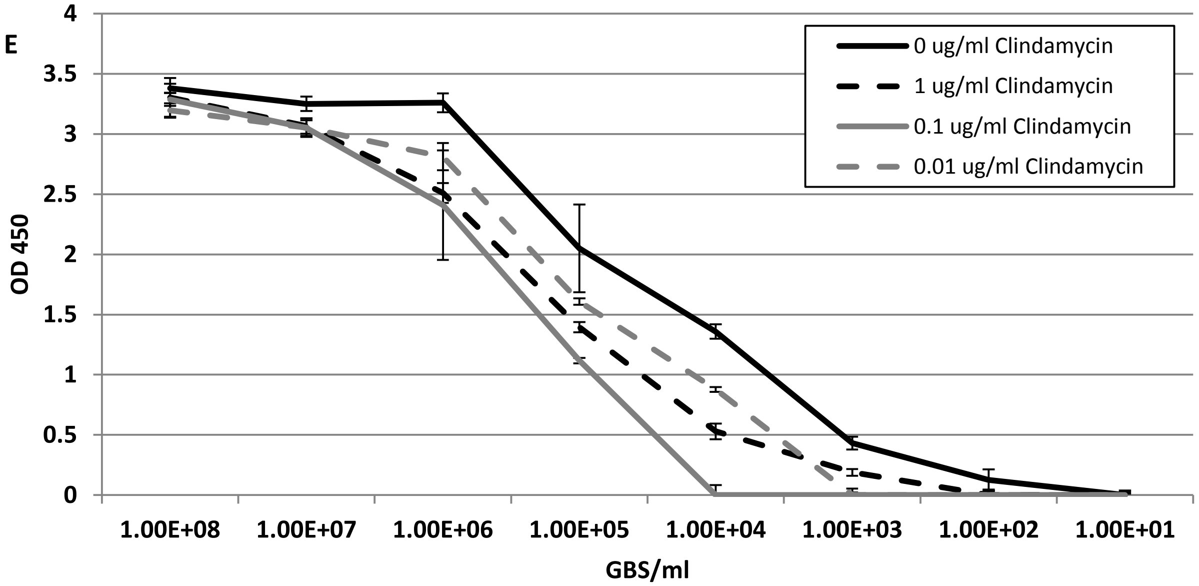
E
OD 450
4
3.5
3
2.5
2
1.5
1
0.5
0
1.00E+08
1.00E+07
1.00E+06
1.00E+05
1.00E+04
1.00E+03
1.00E+02
1.00E+01
GBS/ml
0 ug/ml Clindamycin
1 ug/ml Clindamycin
0.1 ug/ml Clindamycin
0.01 ug/ml Clindamycin

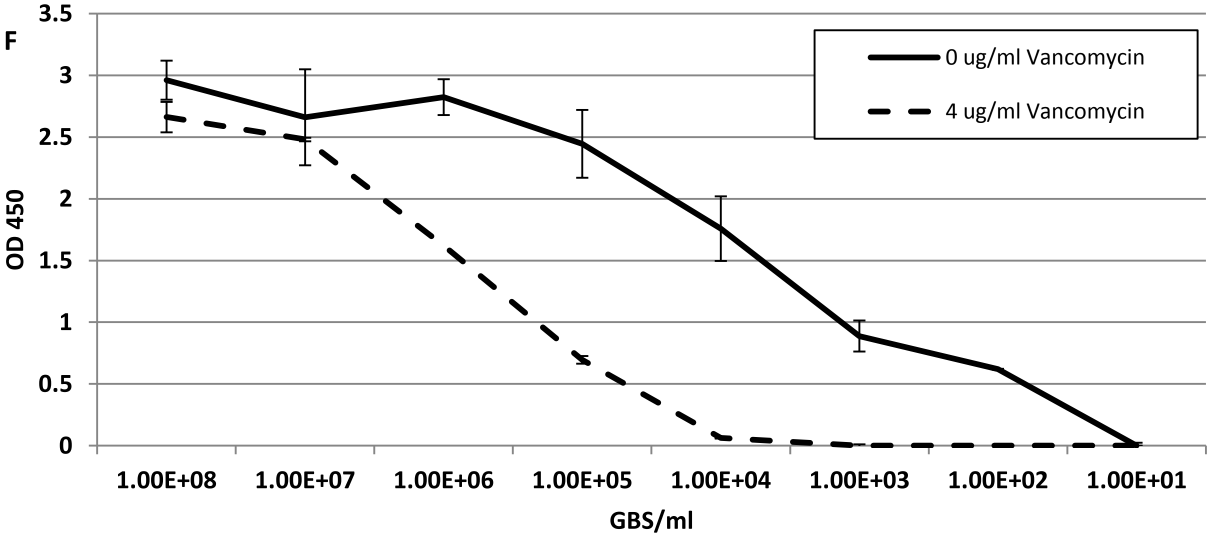
F
OD 450
3.5
3
2.5
2
1.5
1
0.5
0
1.00E+08
1.00E+07
1.00E+06
1.00E+05
1.00E+04
1.00E+03
1.00E+02
1.00E+01
GBS/ml
0 ug/ml Vancomycin
4 ug/ml Vancomycin

G

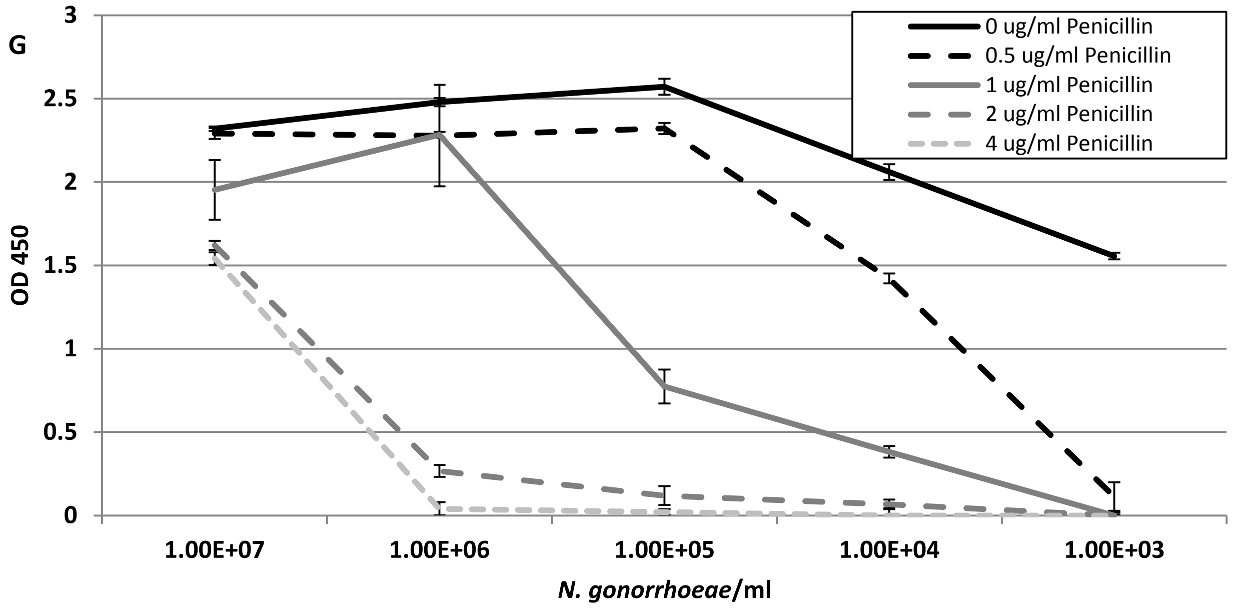

0 ug/ml Penicillin
0.5 ug/ml Penicillin
1 ug/ml Penicillin
2 ug/ml Penicillin
4 ug/ml Penicillin
OD 450
3
2.5
2
1.5
1
0.5
0
1.00E+07
1.00E+06
1.00E+05
1.00E+04
1.00E+03
N. gonorrhoeae/ml

H

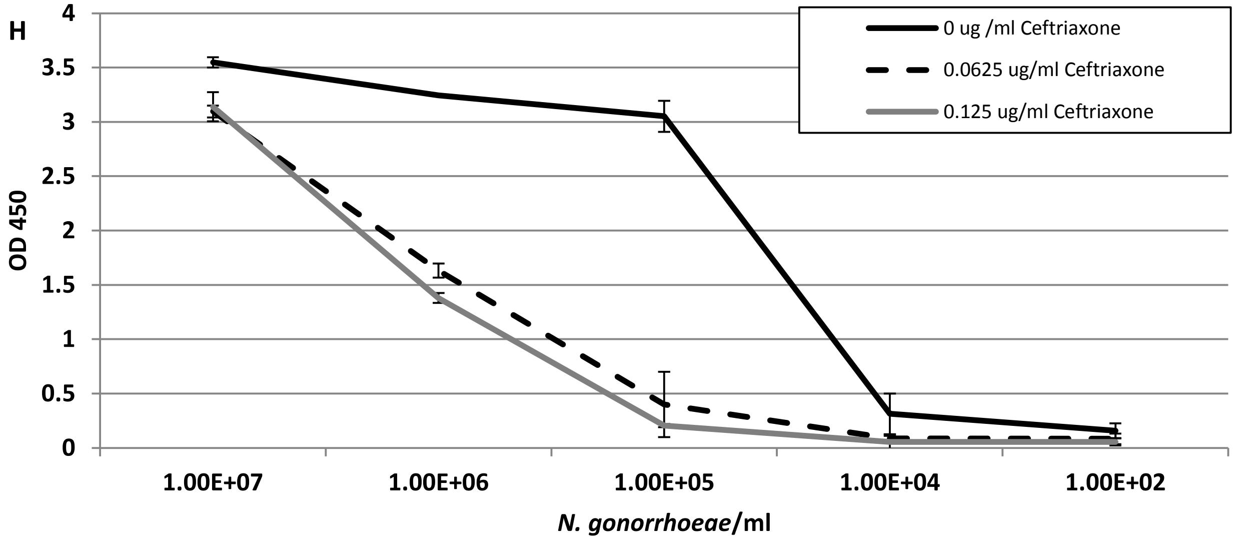

0 ug /ml Ceftriaxone
0.0625 ug/ml Ceftriaxone
0.125 ug/ml Ceftriaxone
OD 450
4
3.5
3
2.5
2
1.5
1
0.5
0
1.00E+07
1.00E+06
1.00E+05
1.00E+04
1.00E+02
N. gonorrhoeae/ml

Supplemental Figure 2 A-H. Demonstration of bacterial resistance to selected antibiotics. After preparing a 0.5 McFarland in Fastidious Broth and then diluting bacterial isolates out, antibiotics were added to the wells giving the final concentrations indicated. A clinical isolate of GBS sensitive to penicillin (strain 12386) was diluted in the presence of either cefazolin at 0, 0.5, 0.05, or 0.005 μg/ml (Panel A), 0, 0.25, 0.025, or 0.0025 μg/ml clindamycin (Panel B), 0, 0.25, 0.025, or 0.0025 μg/ml erythromycin (Panel C). In Panels D and E, a clinical isolate of GBS sensitive to erythromycin but resistant to clindamycin was diluted in the presence of either 0, 0.25, 0.025, or 0.0025 μg/ml erythromycin (Panel D), or 0, 1, 0.1, or 0.01 μg/ml clindamycin (Panel E). Panel F shows a vancomycin-resistant strain of *E. faecalis*, strain 51299, diluted in either 0 or 4 μg/ml vancomycin. In Panel G, a penicillin-resistant strain of *N. gonorrhoeae*, strain 31426, was diluted in the presence of 0, 0.5, 1, 2, or 4 μg/ml penicillin. Finally, Panel H shows a ceftriaxone-sensitive strain of *N. gonorrhoeae*, strain 1279, diluted in the presence of 0, 0.0625 or 0.125 μg/ml ceftriaxone. Incubations were for 6 hours for all studies, except those involving *N. gonorrhoeae*, which were overnight. Each test was performed in triplicate and data shown are representative of three individual experiments. Bacterial concentrations were confirmed by CFUs following plating on agar for 24-48 hours.
